# 血小板线粒体在多发性骨髓瘤细胞增殖与代谢中的作用初探

**DOI:** 10.3760/cma.j.cn121090-20250616-00278

**Published:** 2026-02

**Authors:** 柳芸 张, 云会 向, 艳英 李, 娟 张

**Affiliations:** 1 四川省人民医院临床医学检验中心及遗传性疾病四川省重点实验室，成都 610072 Department of Laboratory Medicine and the Genetic Diseases Key Laboraory of Sichuan Province, Sichuan Provincial People's Hospital, Chengdu 610072, China; 2 四川国际旅行卫生保健中心（成都海关口岸门诊部）口岸疫病疫情监测四川省重点实验室，成都 610041 Department of Laboratory Medicine and Key Laboratory of Port Epidemic Surveillance in Sichuan Province, Sichuan International Travel and Healthcare Center（Chengdu Customs District Port Clinic）, Chengdu 610041, China

**Keywords:** 多发性骨髓瘤, 血小板, 线粒体呼吸, 代谢重编程, 线粒体动力学, Multiple myeloma, Platelets, Mitochondrial respiration, Metabolic reprogramming, Mitochondrial dynamics

## Abstract

**目的:**

分析多发性骨髓瘤（MM）患者的血小板（PLT）及其线粒体特征，并探讨PLT线粒体呼吸对MM细胞增殖、代谢和线粒体动力学的影响。

**方法:**

收集2020年1月至2023年12月四川省人民医院健康志愿者及新诊断MM（NDMM）患者外周血并分离PLT，通过扫描电镜、透射电镜和流式细胞术评估PLT活化状态和线粒体活性氧水平，采用酶联免疫吸附试验（ELISA）检测血清中PLT相关因子表达水平。将健康志愿者未处理的PLT、鱼藤酮或寡霉素预处理PLT分别与MM细胞（RPMI 8226和U266细胞系）共培养。通过CCK-8检测MM细胞增殖，实时荧光定量PCR（qPCR）检测MM细胞中代谢与线粒体动力学相关基因的mRNA表达水平。Western blot检测动力学相关蛋白1（Drp1）及其磷酸化蛋白表达水平。

**结果:**

与健康志愿者相比，MM患者的PLT活化标志物CD41/CD61表达升高［（2.10±1.15）％对（0.22±0.19）％，*P*＝0.048］，CD42b表达下降［（52.80±8.73）％对（74.58±5.11）％，*P*＝0.020］，且PLT线粒体活性氧水平上升（150.50±17.79对62.45±21.34，*P*＝0.001）；血清因子检测显示，MM患者中白细胞介素（IL）-34、血小板因子4（PF4）表达下调，碱性成纤维细胞生长因子（bFGF）、胰岛素样生长因子-1（IGF-1）、IL-6、P-选择素、血小板衍生生长因子（PDGF）和转化生长因子-β1（TGF-β1）表达均上调（*P*值均<0.05），而血管内皮生长因子（VEGF）水平差异无统计学意义（*P*＝0.086）。体外共培养实验表明，与PLT共培养48 h可促进MM细胞增殖，而经鱼藤酮或寡霉素预处理的PLT则丧失促增殖作用（*P*值均<0.001）。qPCR结果显示，共培养后MM细胞代谢相关基因柠檬酸合酶（CS）、乳酸脱氢酶（LDHA）及线粒体动力学相关基因动力蛋白-1样蛋白（DNM1L）、线粒体分裂蛋白1（FIS1）mRNA表达水平均升高（*P*值均<0.05）。Drp1抑制剂Mdivi-1预处理可抑制MM细胞DNM1L mRNA表达（0.75±0.16对1.00±0.09，*P*＝0.002），而与PLT共培养后可逆转抑制作用（1.02±0.13对0.75±0.16，*P*＝0.007）。Western blot结果显示，与PLT共培养后，U266细胞系中p-Drp1 Ser616蛋白表达水平升高（*P*<0.05）。

**结论:**

体外实验提示，PLT及其线粒体呼吸功能可能参与调控MM细胞的增殖、代谢重编程及线粒体动力学过程。然而，其在体内环境及临床实践中的相关性与适用性仍需通过更多临床前及临床研究加以验证。

## Abstract (en)

**Objective:**

To analyze platelet（PLT）characteristics and mitochondrial features in patients with multiple myeloma（MM）, and to investigate the impact of PLT mitochondrial respiration on MM cell proliferation, metabolism, and mitochondrial dynamics.

**Methods:**

Peripheral blood was collected from healthy volunteers and newly diagnosed MM（NDMM）patients at Sichuan Provincial People's Hospital between January 2020 and December 2023, and PLTs were isolated. PLT activation and mitochondrial reactive oxygen species（ROS）levels were assessed by scanning and transmission electron microscopy and flow cytometry. Serum levels of PLT-related factors were measured by enzyme-linked immunosorbent assay（ELISA）. MM cell lines（RPMI 8226 and U266）were co-cultured with untreated PLTs from healthy volunteers or with PLTs pretreated with rotenone or oligomycin. MM cell proliferation was assessed by the CCK-8 assay. mRNA expression of metabolism- and mitochondrial dynamics-related genes in MM cells was quantified by real-time quantitative PCR（qPCR）. Drp1 and phosphorylated Drp1 were analyzed by Western blot.

**Results:**

Compared with healthy volunteers, MM patients showed increased expression of the PLT activation marker CD41/CD61［（2.10 ± 1.15）％ *vs*（0.22 ± 0.19）％, *P*＝0.048］, decreased CD42b expression［（52.80 ± 8.73）％ *vs*（74.58 ± 5.11）％, *P*＝0.020］, and elevated mitochondrial ROS levels in PLTs（150.50 ± 17.79 *vs* 62.45 ± 21.34, *P*＝0.001）. Serum factor analysis showed reduced levels of interleukin-34（IL-34）and platelet factor 4（PF4）and increased levels of basic fibroblast growth factor（bFGF）, insulin-like growth factor 1（IGF-1）, IL-6, P-selectin, platelet-derived growth factor（PDGF）, and transforming growth factor β1（TGF-β1）in MM patients（all *P*<0.05）, whereas vascular endothelial growth factor（VEGF）levels did not differ significantly（*P*＝0.086）. In vitro co-culture experiments showed that co-culture with PLTs for 48 h promoted MM cell proliferation, whereas PLTs pretreated with rotenone or oligomycin lost this pro-proliferative effect（all *P*<0.001）. qPCR showed that co-culture increased mRNA expression of the metabolism-related genes citrate synthase（CS）and lactate dehydrogenase A（LDHA）and the mitochondrial dynamics related genes dynamin-1-like protein（DNM1L）and mitochondrial fission 1（FIS1）in MM cells（all *P*<0.05）. Pretreatment with the Drp1 inhibitor Mdivi-1 inhibited DNM1L mRNA expression in MM cells（0.75 ± 0.16 *vs* 1.00 ± 0.09, *P*＝0.002）; this inhibition was reversed by subsequent co-culture with PLTs（1.02 ± 0.13 *vs* 0.75 ± 0.16, *P*＝0.007）. Western blot analysis showed that co-culture with PLTs increased p-Drp1（Ser616）protein levels in U266 cells（*P*<0.05）.

**Conclusion:**

In vitro experiments suggest that PLTs and their mitochondrial respiratory function may be involved in regulating MM cell proliferation, metabolic reprogramming, and mitochondrial dynamics. However, their relevance and applicability in vivo and in clinical practice require further validation in additional preclinical and clinical studies.

多发性骨髓瘤（MM）是一种具有显著临床和细胞遗传学异质性的血液系统恶性肿瘤。MM的发生与进展高度依赖于骨髓微环境（BME），MM细胞与BME的相互作用，对于疾病获得性耐药及代谢重塑的发生至关重要[1]–[3]。血小板（PLT）由骨髓巨核细胞产生，循环于外周血。静息状态下的PLT通常含有4～6个线粒体，为PLT的聚集、活化及颗粒释放等关键功能提供能量支持[4]–[6]。PLT可通过多种机制影响肿瘤恶性进展的多个环节[7]–[9]。PLT来源的线粒体具有广泛代谢调控功能，能够通过代谢重塑广泛调控受体细胞的功能[10]。研究表明，在伤口愈合这一生理过程中，PLT释放的线粒体可通过增强新生脂肪酸合成等代谢重编程方式，刺激间充质干细胞的促血管生成特性，从而促进组织修复[11]。在肿瘤转移过程中，PLT线粒体可被肿瘤细胞捕获，通过激活PINK1/Parkin-Mfn2通路驱动其代谢转向糖酵解，帮助肿瘤细胞适应氧化应激并增强侵袭能力[9]。

线粒体动力学即线粒体的融合和分裂的动态平衡，在细胞增殖、凋亡，细胞周期及细胞代谢中发挥重要作用[12]。线粒体的分裂过程主要由动力学相关蛋白（Drp1）调控。线粒体分裂过程中，Drp1被激活，从细胞质易位到线粒体表面，与线粒体外膜上的Drp1受体蛋白结合，如线粒体分裂蛋白1（FIS1）[13]–[14]。线粒体动力学与代谢重编程存在紧密的双向调控关系[15]–[16]。在神经元等有丝分裂后细胞中，缺氧引起的酸中毒可通过调控线粒体融合与嵴结构，使细胞仍能维持氧化磷酸化效率与存活[17]。相反，在HeLa及U2OS等细胞系中，线粒体延伸因子1（MIEF1/MiD51）的缺失，会直接损害线粒体呼吸功能并诱发氧化应激，从而促使细胞凋亡或线粒体自噬[18]。在表皮生长因子受体酪氨酸激酶抑制剂耐药的肺癌细胞中的研究进一步揭示，线粒体嵴通过特定蛋白修饰发生重塑，直接驱动了代谢重编程、转移与耐药表型[19]。以上研究结果提示，对线粒体动力学与嵴形态的调控，是细胞实现代谢重塑并决定其适应、存活或疾病进展的关键可塑性节点。

MM患者PLT通常处于高度活化状态，增加了血栓事件的发生风险[20]–[22]。PLT可通过上调MM细胞中白细胞介素（IL）-1β促进MM进展[22]。Natoni等[23]发现PLT通过P-选择素结合到富含唾液酸化结构的MM细胞上，从而抑制NK细胞介导的细胞毒性作用，并促进了MM疾病进展。此外，Kuang等[24]研究发现MM释放至BME中的丝氨酸通过抑制PLT生成而导致的PLT减少症与MM预后不良密切相关。PLT在MM进展中扮演重要角色，但PLT线粒体呼吸作用在MM细胞代谢重塑及线粒体动力学中的具体分子机制，目前仍缺乏深入研究。

基于此，本研究首先通过扫描电镜、透射电镜及流式细胞术研究MM患者PLT及其线粒体特征；其次通过体外共培养模型，初步探究PLT线粒体对MM细胞增殖、代谢及线粒体动力学的影响，旨在揭示PLT线粒体在MM进展中的潜在作用与分子机制。

## 材料与方法

1. 标本收集：本研究已获四川省人民医院伦理委员会的批准［批件号：伦理审查（研）2025年第236号］，所有参与者均签署知情同意书。收集四川省人民医院2020年1月至2023年12月期间101例新诊断MM（NDMM）患者及52名年龄、性别匹配的健康志愿者的空腹血清样本，用于细胞因子检测。MM诊断符合国际骨髓瘤工作组（IMWG）标准，并排除合并其他肿瘤、活动性感染或严重脏器功能不全者。两组基线人口学特征差异均无统计学意义（*P*值均>0.05）。此外，分别收集3例MM患者与3名健康志愿者的抗凝全血用于PLT电镜形态学观察；另外采集3例MM与3名健康志愿者的抗凝全血用于PLT活化率检测；4例MM与4名健康志愿者的血液标本用于PLT线粒体活性氧检测。

2. 细胞培养：人MM细胞系RPMI 8226和U266购自武汉普诺赛生命科技有限公司。细胞在37 °C、5％ CO_2_条件下培养，RPMI 8226细胞和U266细胞均使用其专用培养基（武汉普诺赛生命科技有限公司）。本研究中，功能实验（如共培养、CCK-8增殖实验）主要在RPMI 8226细胞系中完成，以重点探索表型；而关键分子验证［如实时荧光定量PCR（qPCR）、Western blot］则在2个细胞系中均进行验证以确认趋势。

3. PLT形态的扫描电镜和透射电镜观察：收集3例NDMM患者及3名健康志愿者EDTA-K2抗凝的空腹全血标本2 ml，于4 h内分离纯化PLT。将纯化PLT样品轻柔粘附于导电胶，经离子溅射喷镀后通过扫描电镜观察。分离的PLT经3％戊二醛前固定和1％锇酸后固定，再用丙酮脱水（浓度梯度依次为30％、50％、70％、80％、90％、95％、100％）。脱水后样品依次经过脱水剂和环氧树脂渗透液（比例3∶1、1∶1、1∶3，各1 h）渗透、包埋，制备超薄切片（厚度50～70 nm），经醋酸铀和柠檬酸铅（北京中镜科仪技术有限公司）双染色，室温下染色15～20 min后，使用JEM-1400PLUS透射电镜观察。

4. 流式细胞术检测PLT活化及线粒体活性氧水平：分别收集NDMM患者和健康志愿者的枸橼酸钠抗凝外周血3 ml，取5 µl全血，加入PerCP抗人CD42b和FITC抗人CD41/CD61抗体（美国Biolegend公司）各5 µl，室温避光孵育20 min，经BD Cytofix™缓冲液固定后上机检测。另采集4例NDMM患者和4名健康志愿者的全血3 ml，通过差速离心分离PLT。取PLT悬液与10 µmol/L MitoSOX Red（美国MedChemExpress公司）避光孵育30 min，PBS洗涤后，加入PerCP抗人CD42b抗体5 µl室温避光孵育20 min，洗涤重悬后上机检测。流式细胞术实验数据均采用FlowJo v10.8.1软件分析。

5. 酶联免疫吸附试验（ELISA）检测细胞因子表达：收集101例NDMM患者和52名健康志愿者的空腹血清样本进行细胞因子检测。ELISA试剂盒均购自上海茁彩生物科技有限公司，严格遵循碱性成纤维细胞生长因子（bFGF）（货号：ZC-33488）、胰岛素样生长因子-1（IGF-1）（货号：ZC-35433）、IL-6（货号：ZC-32446）、血管内皮生长因子（VEGF）（货号：ZC-35248）、P-选择素（货号：ZC-32038）、IL-34（货号：ZC-54397）、血小板衍生生长因子（PDGF）（货号：ZC-35385）、血小板因子4（PF4）（货号：ZC-35351）和转化生长因子-β1（TGF-β1）（货号：ZC-35791）各试剂盒说明书进行操作。

6. CCK-8检测PLT线粒体功能对MM细胞增殖的影响：收集3名健康志愿者含枸橼酸钠抗凝的新鲜无菌外周全血标本5 ml各1管，差速离心分离PLT。实验共设5组：①对照组（单独培养RPMI 8226细胞）；②PLT 1×（MM细胞与PLT共培养比例1∶100）；③PLT 2×（共培养比例1∶200）；④鱼藤酮-PLT 2×（MM细胞与经2 µmol/L线粒体复合物Ⅰ抑制剂鱼藤酮处理2.5 h的PLT按1∶200比例共培养）；⑤寡霉素-PLT 2×组（MM细胞与经3 µmol/L线粒体复合物Ⅴ抑制剂寡霉素处理1 h后的PLT按1∶200比例共培养）。鱼藤酮和寡霉素购自美国ApexBio Technology公司。共培养比例参考前期预实验及相关文献设定[22]。准备3个96孔板，每组设5个复孔，分别于共培养12、24、48 h时，每孔加入10 µl CCK-8溶液，孵育2 h后，使用酶标仪在450 nm（主波长）和650 nm（参比波长）下测定吸光度值。

7. qPCR检测代谢及动力学相关基因表达：实验分组：对照组、PLT 2×、鱼藤酮-PLT 2×、寡霉素-PLT 2×组。共培养48 h后，使用8 µl APC抗人CD138抗体（美国Biolegend公司）标记MM细胞，通过BD FACSAria Ⅲ流式分选仪获得MM细胞，其中RPMI 8226细胞流式细胞术分选圈门如图1所示，U266细胞分选策略类似。按试剂盒说明书，使用SteadyPure快速RNA提取试剂盒（湖南艾科瑞生物工程有限公司）提取总RNA。使用反转录试剂盒Ⅱ（预混gDNA消化酶）（美国MedChemExpress公司）进行逆转录。qPCR采用SYBR Green qPCR预混液（通用型）（美国MedChemExpress公司）。以GAPDH为内参，采用2^−ΔΔCt^法计算柠檬酸合酶（CS）、乳酸脱氢酶（LDHA）、脂肪酸合酶（FASN）、ATP柠檬酸裂解酶（ACLY）、动力学蛋白1样（DNM1L）和FIS1基因mRNA相对表达，其中CS与LDHA分别与氧化磷酸化和有氧糖酵解途径密切相关，而FASN和ACLY与脂肪酸合成密切相关。引物序列均由北京擎科生物科技股份有限公司合成（表1）。

**图1:**
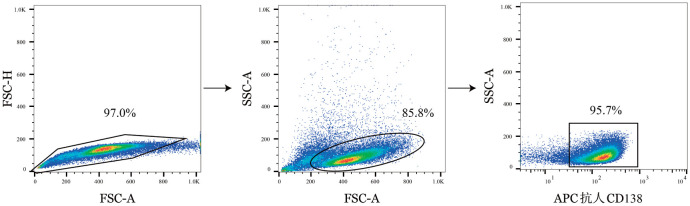
多发性骨髓瘤细胞流式细胞术分选圈门图

**表1:** 实时荧光定量PCR检测基因的引物序列

基因名称	正向引物（5′-3′）	反向引物（5′-3′）
GAPDH	GGAGCGAGATCCCTCCAAAAT	GGCTGTTGTCATACTTCTCATGG
CS	TTGGGGCCATTGACTCTAAC	GCTGCAAAGGACAGGTAAGG
LDHA	ATGGCAACTCTAAAGGATCAGC	CCAACCCCAACAACTGTAATCT
FASN	CTTCCGAGATTCCATCCTACGC	TGGCAGTCAGGCTCACAAACG
ACLY	TGCTCGATTATGCACTGGAAGT	ATGAACCCCATACTCCTTCCCAG
DNM1L	CTGCCTCAAATCGTCGTAGTG	GAGGTCTCCGGGTGACAATTC
FIS1	GTCCAAGAGCACGCAGTTTG	ATGCCTTTACGGATGTCATCATT

**注** GAPDH：甘油醛-3-磷酸脱氢酶；CS：柠檬酸合酶；LDHA：乳酸脱氢酶；FASN：脂肪酸合酶；ACLY：ATP柠檬酸裂解酶；DNM1L：动力学蛋白1样；FIS1：线粒体分裂蛋白1

8. Western blot检测动力学相关蛋白表达：实验分为对照组和PLT 2×组。共培养48 h后收集细胞，加入含抑制剂的RIPA裂解液（北京索莱宝科技有限公司）冰上裂解5～10 min，超声破碎细胞后4 °C，12 000 ×*g*离心6 min取上清。按试剂盒说明书，用BCA蛋白浓度测定试剂盒（北京兰杰柯科技有限公司）定量蛋白浓度。经PAGE凝胶快速制备试剂盒12.5％（上海雅酶生物医药科技有限公司）电泳分离，转至硝酸纤维素膜。用5％脱脂牛奶室温封闭1 h，加入一抗4 °C孵育过夜。抗体为：Phospho-Drp1（Ser616）（1∶2 000）、Phospho-Drp1（Ser637）（1∶2 000）（美国Affinity Biosciences公司）；Drp1（C-terminal）（1∶5 000）、GAPDH（1∶10 000）、HRP-conjugated Goat Anti-Rabbit IgG（H+L）（1∶10 000）、HRP-conjugated Goat Anti-Mouse IgG（H+L）抗体（1∶10 000）（武汉三鹰生物技术有限公司）。洗涤后与相应HRP标记的二抗室温孵育1 h，ECL发光显影。以GAPDH为内参，用Image J软件进行Western blot法蛋白条带灰度值计算。实验在技术重复条件下完成，结果代表初步趋势。

9. 统计学处理：采用Graphpad prism 9进行统计分析与作图。符合正态分布的计量资料以*x*±*s*表示，组间比较采用独立样本*t*检验；不符合正态分布的计量资料以中位数（四分位间距）［*M*（*Q*_1_，*Q*_3_）］表示，组间比较采用Mann-Whitney *U*检验。多组间比较，若数据符合正态分布且方差齐，采用单因素方差分析（one-way ANOVA）；否则采用非参数的Kruskal-Wallis检验。以*P*<0.05为差异具有统计学意义。

## 结果

1. MM患者的PLT过度激活：为探索MM患者PLT及其线粒体的特征，采用扫描电镜和透射电镜观察PLT形态。扫描电镜观察显示，健康志愿者的PLT形状相对规则，而MM患者的PLT形状不规则且高度可变；透射电镜观察结果显示，健康志愿者的PLT形态结构正常，线粒体可见，内质网粗糙，颗粒状结构清晰，线粒体嵴清晰。而MM患者的PLT表现为空泡化、偶有脂滴和线粒体轻度肿胀（图2）。进一步通过流式细胞术检测PLT表面标志物CD42b和CD41/CD61的表达以及线粒体活性氧水平。结果显示，与健康志愿者相比，MM患者的CD41/CD61表达升高［（2.10±1.15）％对（0.22±0.19）％，*P*＝0.048］，而CD42b表达下降［（52.80±8.73）％对（74.58±5.11）％，*P*＝0.020］，提示MM患者体内PLT活化增强。同时，MM患者PLT线粒体活性氧水平显著高于健康志愿者（150.50±17.79对62.45±21.34，*P*＝0.001），这与透射电镜观察到的线粒体轻度肿胀一致。此外，通过ELISA检测血清细胞因子发现，与健康志愿者相比，MM患者中IL-34、PF4表达下调，bFGF、IGF-1、IL-6、PDGF、P-选择素、TGF-β1表达均上调（*P*值均<0.05），而VEGF水平差异无统计学意义（*P*＝0.086，表2）。

**图2:**
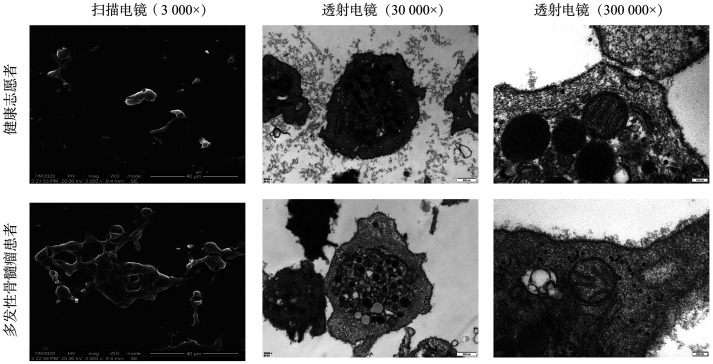
扫描电镜及透射电镜观察多发性骨髓瘤患者与健康志愿者的血小板形态结构

**表2:** 酶联免疫吸附试验检测多发性骨髓瘤患者与健康志愿者血清样本的细胞因子比较［*M*（*Q*_1_，*Q*_3_）］

细胞因子类型	多发性骨髓瘤患者（101例）	健康志愿者（52名）	*z*值	*P*值
bFGF（pg/ml）	29.30（18.41，38.32）	18.96（12.24，29.72）	−3.90	<0.001
IGF-1（ng/ml）	62.60（41.36，79.93）	43.44（31.17，62.38）	−3.87	<0.001
IL-6（pg/ml）	8.72（6.50，12.43）	6.26（4.38，8.46）	−3.58	<0.001
VEGF（pg/ml）	103.10（76.59，135.90）	95.87（62.00，119.50）	−1.72	0.086
P-选择素（ng/ml）	3.23（1.90，4.64）	2.40（1.20，3.49）	−2.20	0.027
IL-34（pg/ml）	323.90（251.10，402.10）	378.60（298.50，486.50）	−2.42	0.015
PDGF（ng/ml）	47.73（37.43，62.85）	42.80（28.68，51.84）	−2.65	0.008
PF4（ng/ml）	4.10（2.81，5.72）	6.03（4.27，7.17）	−4.63	<0.001
TGF-β1（ng/ml）	78.94（60.62，98.35）	65.66（51.78，81.98）	−3.26	0.001

**注** bFGF：碱性成纤维细胞生长因子；IGF-1：胰岛素样生长因子-1；IL：白细胞介素；VEGF：血管内皮生长因子；PDGF：血小板衍生生长因子；PF4：血小板因子4；TGF-β1：转化生长因子-β1

2. PLT线粒体呼吸促进MM细胞增殖：通过体外共培养实验研究PLT线粒体呼吸对MM细胞增殖的影响。将RPMI 8226细胞单独培养或与经不同处理的PLT共培养12 h、24 h、48 h后，CCK-8法检测MM细胞增殖。结果显示，线粒体呼吸功能完整的PLT可促进MM细胞增殖，而经过鱼藤酮或寡霉素处理致线粒体呼吸受损的PLT则无此作用，甚至抑制细胞增殖（表3）。培养至12 h时，所有共培养组与对照组间差异均无统计学意义（*P*值均>0.05）。培养至24 h时，PLT 1×组的相对细胞增殖量高于对照组（0.115±0.009对0.093±0.008，*P*＝0.003），而PLT 2×组（0.105±0.001）与对照组的差异无统计学意义（*P*＝0.340）。然而，培养延长至48 h时，两种比例的PLT共培养均展现出显著的促增殖效应：PLT 1×组（0.215±0.010）及PLT 2×组（0.267±0.017）均高于对照组（0.184±0.010，*P*值均<0.001），且以1∶200比例（PLT 2×）的促进作用最为明显。此外，与PLT 2×组相比，鱼藤酮-PLT 2×组和寡霉素-PLT 2×组在48 h的细胞增殖均被抑制（均*P*<0.001）。鉴于1∶200比例共培养48 h时促增殖效应最明显，后续实验均采用此条件。

**表3:** 血小板（PLT）线粒体呼吸功能对多发性骨髓瘤（MM）细胞增殖的影响（相对细胞增殖量，*x*±*s*）

组别	培养时间		
	12 h	24 h	48 h
对照组	0.003±0.0002	0.093±0.008	0.184±0.010
PLT 1×	0.003±0.0001	0.115±0.009	0.215±0.010
PLT 2×	0.003±0.0003	0.105±0.001	0.267±0.017
鱼藤酮-PLT 2×	0.017±0.001	0.034±0.014	0.173±0.004
寡霉素-PLT 2×	0.003±0.0004	0.067±0.018	0.133±0.009

*P*_1_值	>0.05	0.003	<0.001
*P*_2_值	>0.05	0.340	<0.001
*P*_3_值	0.275	<0.001	<0.001
*P*_4_值	>0.05	<0.001	<0.001

**注** 对照组：单独培养的MM细胞（RPMI 8226细胞系）；PLT 1×：MM细胞与PLT以1∶100的比例共培养；PLT 2×：MM细胞与PLT以1∶200的比例共培养；鱼藤酮-PLT 2×：MM细胞与经2 µmol/L鱼藤酮处理2.5 h后的PLT以1∶200的比例共培养；寡霉素-PLT 2×：MM细胞与经3 µmol/L寡霉素处理1 h后的PLT以1∶200的比例共培养；*P*_1_：对照组与PLT 1×组比较；*P*_2_：对照组与PLT 2×组比较；*P*_3_：PLT 2×组与鱼藤酮-PLT 2×组比较；*P*_4_：PLT 2×组与寡霉素-PLT 2×组比较

3. PLT线粒体呼吸促进MM细胞代谢重编程：为初步探索PLT在MM细胞代谢中的作用，将MM细胞（RPMI 8226或U266细胞系）单独培养或与经不同处理的PLT共培养48 h，随后通过qPCR检测流式细胞术分选后MM细胞中CS、LDHA、FASN、ACLY的mRNA表达水平。结果显示，在RPMI 8226和U266细胞系中，与对照组相比，PLT 2×组CS、LDHA mRNA表达水平均升高；而在RPMI 8226细胞中，鱼藤酮-PLT 2×组和寡霉素-PLT 2×组较PLT 2×组CS、LDHA、FASN、ACLY mRNA表达水平均降低（*P*值均<0.05），在U266细胞系中差异无统计学意义（图3）。

**图3:**
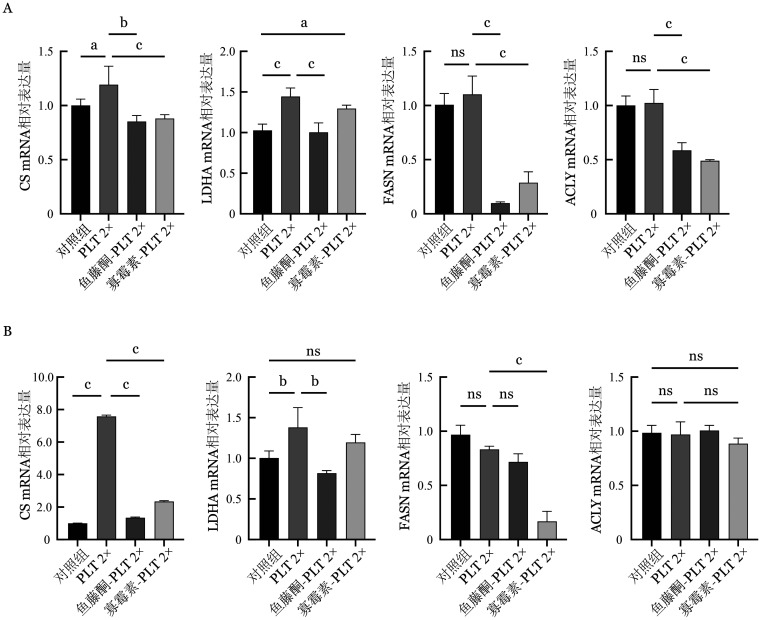
qPCR检测血小板（PLT）共培养对MM细胞代谢重编程的影响：MM细胞CS、LDHA、FASN和ACLY的相对mRNA表达水平 **注** qPCR：实时荧光定量PCR；MM：多发性骨髓瘤；A：RPMI 8226细胞系mRNA表达情况；B：U266细胞系mRNA表达情况；CS：柠檬酸合酶；LDHA：乳酸脱氢酶；FASN：脂肪酸合酶；ACLY：ATP柠檬酸裂解酶；对照组：单独培养的MM细胞（RPMI 8226或U266细胞系）；PLT 2×：MM细胞与PLT以1∶200的比例共培养；鱼藤酮-PLT 2×：MM细胞与经2 µmol/L鱼藤酮处理2.5 h后的PLT以1∶200的比例共培养；寡霉素-PLT 2×：MM细胞与经3 µmol/L寡霉素处理1 h后的PLT以1∶200的比例共培养；^ns^ *P*>0.05；^a^*P*<0.01；^b^ *P*<0.001；^c^ *P*<0.0001

4. PLT线粒体呼吸促进MM细胞线粒体动力学：为探索PLT对MM细胞线粒体动力学的影响，首先通过qPCR检测分裂关键基因DNM1L及FIS1的mRNA表达水平。将MM细胞单独培养或与不同处理的PLT共培养48 h。结果显示，在RPMI 8226细胞系中，与对照组（DNM1L：1.01±0.12；FIS1：1.01±0.19）相比，PLT 2×组FIS1 mRNA表达上调（1.43±0.17，*P*＝0.002），DNM1L mRNA表达差异无统计学意义（*P*＝0.133）。鱼藤酮-PLT 2×组和寡霉素-PLT 2×组与PLT 2×组相比，DNM1L和FIS1 mRNA表达差异均无统计学意义（*P*值均>0.05）。在U266细胞系中，与对照组相比，PLT 2×组DNM1L mRNA（1.17±0.11对1.00±0.04，*P*＝0.011）与FIS1 mRNA（1.27±0.04对1.01±0.17，*P*＝0.024）表达均上调。与PLT 2×组相比，寡霉素-PLT 2×组DNM1L mRNA（1.37±0.05对1.17±0.11，*P*＝0.008）与FIS1 mRNA（2.23±0.12对1.27±0.04，*P*<0.001）表达均进一步上调。

随后通过Western blot检测单独及共培养组的MM细胞Drp1及其最具特征的磷酸化位点p-Drp1 Ser616和p-Drp1 Ser637蛋白表达。结果表明，在U266细胞系中，PLT 2×组较对照组p-Drp1 Ser616蛋白表达增加（*P*<0.05），提示Drp1活化增强，而RPMI 8226细胞系中p-Drp1 Ser616蛋白表达无显著变化；两个细胞系中p-Drp1 Ser637蛋白表达与对照组相比差异均无统计学意义（*P*值均>0.05，图4）。为验证Drp1在PLT介导线粒体分裂中的必要性，采用Drp1特异性抑制剂Mdivi-1（15 µmol/L）预处理RPMI 8226细胞系24 h，再与PLT按照1∶200的比例共培养。结果发现，Mdivi-1预处理抑制MM细胞DNM1L mRNA表达（0.75±0.16对1.00±0.09，*P*＝0.002），而对FIS1 mRNA表达无影响（1.09±0.07对1.01±0.16，*P*＝0.510）。Mdivi-1处理的MM细胞在与PLT共培养后，DNM1L mRNA表达仍可升高至1.02±0.13（*P*＝0.007）。

**图4:**
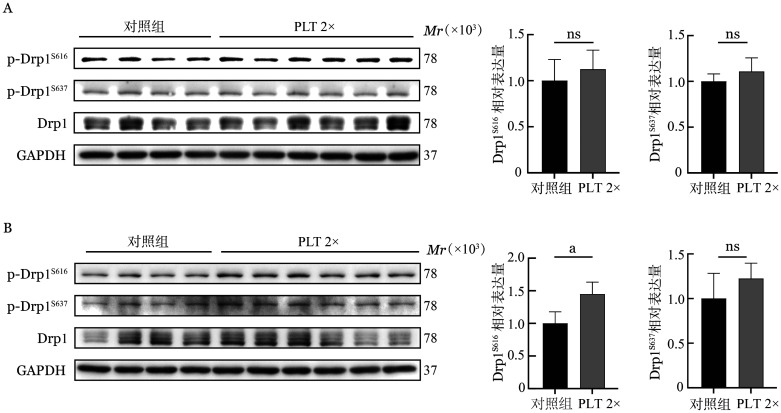
Western blot法检测MM细胞Drp1、p-Drp1 Ser616、p-Drp1 Ser637蛋白表达 **注** MM：多发性骨髓瘤；A：RPMI 8226细胞系蛋白表达情况；B：U266细胞系蛋白表达情况；PLT：血小板；对照组：单独培养的MM细胞（RPMI 8226或U266细胞系）；PLT 2×：MM细胞与PLT以1∶200的比例共培养；^ns^ *P*>0.05；^a^ *P*<0.01

## 讨论

MM进展高度依赖于BME的复杂调控[25]，尽管BME中多种细胞（如T细胞、NK细胞、单核细胞、巨噬细胞）及非细胞成分在支持MM细胞存活、增殖、免疫逃逸、耐药及复发中的作用已被广泛研究[26]，但PLT在该过程的作用，尤其是其线粒体呼吸功能的具体机制，尚未被充分阐明。近年虽有多项研究强调了PLT在MM进展中的重要性[22]–[24]，但其线粒体呼吸是否以及如何参与该过程仍属未知。本研究通过体外共培养体系初步发现，PLT线粒体呼吸可能通过诱导代谢重编程与改变线粒体动力学促进MM细胞增殖。然而，这些结果目前仍属机制探索阶段，其体内的相关性及临床意义尚需进一步验证。

本研究发现MM患者PLT存在线粒体超微结构异常，表现为轻度肿胀、嵴结构模糊，并伴有线粒体活性氧水平显著升高，提示MM患者PLT处于氧化应激状态。然而，该异常究竟源于MM细胞对PLT的逆向调控，还是PLT自身原发功能紊乱，目前尚无定论。体外共培养实验进一步显示，功能性PLT线粒体可显著促进MM细胞增殖；而当使用鱼藤酮或寡霉素部分抑制PLT线粒体呼吸后，MM细胞增殖受到限制，表明PLT线粒体可能为MM细胞提供代谢支持。

此外，MM患者血清细胞因子谱分析显示，多种与PLT活化、肿瘤生长及免疫调节相关的因子水平显著上调，包括bFGF、IGF-1、IL-6、PDGF、P-选择素、TGF-β1。其中，P-选择素是PLT活化的直接标志物，其升高进一步验证了PLT在MM患者体内处于活化状态。上述细胞因子是已知的促肿瘤生长、血管生成或免疫抑制因子[27]，而IL-6也在MM进展和骨病中发挥重要作用[28]。值得注意的是，IL-34和PF4表达水平在MM患者中显著下调，而VEGF水平则未见差异。这提示PLT可能是PDGF、TGF-β1、P-选择素、PF4等细胞因子的重要来源或参与其调控，但PLT活化状态及线粒体功能是否直接影响这些因子的分泌或活性，仍需深入验证。

PLT线粒体呼吸可能参与调控MM细胞的代谢重编程。共培养实验表明，PLT可在体外上调MM细胞氧化磷酸化相关基因CS和有氧糖酵解关键基因LDHA的mRNA水平，而抑制PLT线粒体呼吸可削弱这一效应。这提示PLT线粒体呼吸可能为MM细胞提供了代谢支持。然而，仍需通过测定细胞外酸化率（ECAR）、氧消耗率（OCR）等功能性代谢指标，直接评估PLT线粒体呼吸在MM细胞糖酵解及氧化磷酸化过程中的具体作用。

线粒体动力学与细胞代谢状态密切相关。本研究发现PLT共培养可上调MM细胞中p-Drp1 Ser616位点磷酸化水平，并上调分裂相关基因DNM1L与FIS1的mRNA表达；而使用Drp1抑制剂Mdivi-1处理则降低了MM细胞DNM1L的mRNA表达。多数研究支持Drp1 Ser616磷酸化促进线粒体分裂，但是p-Drp1 Ser637位点的作用仍存在争议[29]–[30]。这些结果提示Drp1依赖的线粒体分裂可能参与PLT诱导的MM细胞代谢适应，但其精确调控机制仍需进一步解析。

本研究从体外水平初步揭示了PLT线粒体呼吸在调控MM细胞增殖、代谢重编程和线粒体动力学中的潜在作用，但该发现不能直接外推至临床输血实践。由于缺乏体内实验验证，且未充分考虑PLT在体内的存活、清除及其与肿瘤免疫微环境的复杂相互作用等因素，PLT输注与MM进展之间的临床关联仍未明确。尽管一项基于骨肉瘤细胞系K7的小鼠皮下成瘤实验研究提示，线粒体呼吸受损的PLT可增强抗肿瘤效应[9]，但其预处理方式与临床输血存在显著差异，使其难以直接转化应用于临床。目前尚无流行病学证据支持PLT输注会加速MM进展；相反，PLT减少症是MM患者常见的临床并发症，依据现行指南规范输注仍是重要治疗手段。若未来研究确证PLT线粒体呼吸具有促进肿瘤进展的作用，则开发“去线粒体”或功能调控型PLT可能成为兼顾止血与肿瘤治疗安全性的新策略。
